# Use of inhaled corticosteroids for the prevention and/or treatment of bronchopulmonary dysplasia in preterm infants: a systematic review protocol

**DOI:** 10.1186/s13643-015-0108-1

**Published:** 2015-09-25

**Authors:** Eric S. Shinwell, Igor Portnov, Joerg Meerpohl, Tanja Karen, Dirk Bassler

**Affiliations:** Department of Neonatology, Ziv Medical Center, Rambam Street, Tsfat, 13100 Israel; Cochrane Germany, Medical Center, University of Freiburg, Berliner Allee 29, 79110 Freiburg, Germany; Department of Neonatology, University Hospital Zurich, Frauenklinikstr. 10, 8091 Zurich, Switzerland

**Keywords:** Preterm infant, Bronchopulmonary dysplasia, Inhaled corticosteroids, Corticosteroids, Prevention

## Abstract

**Background:**

Inhaled steroids have been studied for both prevention and treatment of bronchopulmonary dysplasia (BPD). Results have been inconsistent. Recently, a large randomized controlled trial (RCT) has been reported.

**Methods/design:**

We will perform a comprehensive systematic literature search for randomized and quasi-randomized controlled trials that studied the efficacy and safety of inhaled corticosteroids administered to preterm infants (22–36 weeks) for either the prevention or treatment of BPD diagnosed by both clinical and physiological outcome criteria. We will assess potential risk of bias for each RCT meeting our selection criteria using the Cochrane risk of bias tool for RCTs. The primary outcome of interest will be mortality or BPD or both at 28 days postnatal age or 36 weeks postmenstrual age. Pooled estimates will be calculated using RevMan software with a random effects model as primary analysis. We will assess the quality of evidence using the Grading of Recommendations Assessment, Development and Evaluation (GRADE) approach.

**Discussion:**

Meta-analytic estimates of eligible RCTs, potentially including a new large RCT, may significantly influence neonatal practice in the prevention and treatment of respiratory problems in preterm infants.

**Systematic review registration:**

PROSPEROCRD42015019628

## Background

Bronchopulmonary dysplasia (BPD) remains a major cause of mortality and morbidity in extremely low birth weight (ELBW) infants and is associated with an increased risk for neurodevelopmental impairment in later years [1]. The pathogenesis of BPD is closely linked to inflammatory processes within the immature lung [2–4]. Due to their anti-inflammatory properties, corticosteroids have been and are still widely used for both the prevention and treatment of BPD in preterm infants [4]. Early systemic use of corticosteroids is associated with significant adverse effects on growth and neurodevelopmental outcome with increased incidence of cerebral palsy. Inhaled administration is an attractive alternative that may offer clinical efficacy without incurring adverse effects [3]. This intervention has been studied in a number of randomized controlled trials (RCTs) and other studies [4]. They have employed a variety of drugs, at a wide range of doses and administered over a wide range of periods of time, representing both early prevention and later treatment or prolonged duration covering both options. The main problem of these reports is the small numbers of infants included, and overall, the numbers have been insufficient to establish a safety profile. Moreover, the results have shown either modest or absent efficacy. Recently, a large multicenter trial has been conducted that may significantly alter the findings of a previously published meta-analysis [5].

Previous meta-analyses have attempted to separate studies of prevention and treatment, despite significant overlap in ages at administration of the inhaled steroid [6–9]. Shah et al. reported a meta-analysis of studies of “early” postnatal inhaled steroids, defined as administration that was started before the age of 2 weeks. This analysis included 7 trials with 492 infants, although the primary and secondary outcome variables were only reported in 5 of the 7 trials including 429 infants [6]. The duration of the study intervention varied from 10 days to 4 weeks. Onland et al. conducted a meta-analysis of “late” studies defined as treatment started after 7 days of age [8]. As the entry criteria of these two analyses overlapped and both included studies that started between age 1 and 2 weeks, one study met inclusion criteria for both analyses. The “late” analysis included 8 studies and 232 infants. However, most of the main outcome variables were reported in only a very small number of the studies with very few infants.

In addition to these two analyses, there are two additional analyses comparing inhaled and systemic steroids for either prevention or treatment of BPD. It is not possible to include the inhaled arm of these studies in a meta-analysis of early or late inhaled steroids as there is no appropriate control group.

Accordingly, this systematic review offers the likelihood of useful practical information for practitioners considering the role of inhaled steroids for preterm infants at risk for BPD. This systematic review will add a single very large study that includes more infants than all the above studies together and by combining both approaches, prevention and treatment, and including preterm infants from birth onwards, it is hoped that the sufficient sample size will answer this important question.

## Methods/design

This protocol was prepared according to the PRISMA-P guideline of 2015, and the systematic review will be reported according to PRISMA guidelines (2009) [10, 11].

### Objective

The objective of this study is to assess the efficacy and safety of inhaled corticosteroids administered to preterm infants for either the prevention or treatment of BPD.

### Types of studies

Randomized controlled trials and quasi-randomized controlled trials (quasi-RCTs) will be eligible for this review.

### Type of participants

This review will include studies whose participants were preterm infants (gestational age 22-36 weeks) at risk for BPD including both ventilated and non-ventilated infants. BPD may be diagnosed by differing criteria that include requirement for supplemental oxygen at 28 days of life or 36 weeks corrected gestational age. Alternatives include a physiological definition involving a room-air challenge at 36 weeks or even the National Institute of Health (NIH) criteria [12, 13].

### Types of interventions

The types of interventions include any inhaled corticosteroid versus control (placebo or no treatment) at any dose and any duration of treatment. Corticosteroids may be administered either by a metered-dose inhaler or by nebulization. Trials of systemic corticosteroids and corticosteroids administered by direct tracheal instillation will not be included in this review.

### Types of outcomes

The primary outcomes will be mortality or BPD or both at 28 days postnatal age (PNA) or 36 weeks postmenstrual age (PMA). Each of these outcomes will be analyzed as a dichotomous variable.

Secondary outcomes will include the individual components of the primary outcome, duration of mechanical ventilation, need for reintubation after end of study treatment, failure to extubate at day 7 and day 14 after initiating therapy, and the supplemental fractional concentration of inspired oxygen (FiO_2_) at both age 28 days and 36 weeks. Additional secondary outcomes include duration of oxygen and positive respiratory pressure support, mortality at hospital discharge, the use of systemic steroids, incidence of persistent ductus arteriosus, incidence of persistent ductus arteriosus requiring drug treatment or surgery, necrotizing enterocolitis (≥bell stage 2), sepsis confirmed by blood culture or clinically suspected, intraventricular hemorrhage (IVH) of any grade, IVH grades 3 and 4, periventricular leukomalacia (PVL), and retinopathy of prematurity (ROP) of any grade and ROP requiring treatment. Long-term neurodevelopmental sequelae, assessed at least after 1-year corrected gestational age and up to 24 months of age including cerebral palsy, blindness, and deafness, and Bayley’s Scale of Infant Development (Mental Development Index (MDI)) will also be assessed.

Additional secondary outcomes will include adverse effects known to be related to steroid therapy including hyperglycemia, hypertension, cardiomyopathy, infections, gastrointestinal hemorrhage or perforation, growth delay, cataracts, tongue hypertrophy, nephrocalcinosis, and suppression of the hypothalamic-pituitary-adrenal axis as defined by an abnormal ACTH stimulation test. Each adverse event will be considered as a separate outcome.

### Search methods

#### Electronic searches

Relevant studies will be identified by searching PubMed.gov of the US National Library of Medicine (Medline from 1966 onwards), the Cochrane Library, EMBASE (from 1974 onwards), and the Cumulative Index to Nursing and Allied Health Literature (CINAHL from 1982 onwards). Search strategies are shown in Appendix 1

#### Searching for other sources

We will also search the trial registers www.Clinicaltrials.gov, www.controlled-trials.com, and www.who.int/trialsearch, lists of references from relevant studies, and abstracts from the proceedings of relevant academic meetings, including the Pediatric Academic Societies and the European Society for Pediatric Research.

### Data collection and analysis

#### Selection of studies

References identified via the literature search will be screened by two of the authors, and disagreement will be resolved either by discussion or with the aid of an additional reviewer. Studies in all languages and only published as abstracts may potentially be included. Studies that were published from 1995 onwards will be included and will include all preterm infants at risk for developing BPD.

### Data extraction and management

Data will be extracted to piloted data extraction forms and entered into Review Manager (RevMan 5.3) by one of the reviewers and checked by a second reviewer. Discrepancies in data extraction or entry will be resolved by a discussion with a third reviewer.

We will extract the following information: characteristics of the study (e.g., language of publication, year of publication), characteristics of the study population, description of the intervention and comparisons, outcome measures and measurement tools, and results.

### Assessment of risk of bias in included studies

The assessment of risk of bias will be performed by two reviewers independently considering the following domains according to the Cochrane risk of bias tool: sequence generation, allocation concealment, blinding (of participants, personnel, and outcome assessors), incomplete outcome data, selective outcome reporting, and other sources of bias for the RCTs. According to the Cochrane Handbook, these items will be described as having a “low,” “high,” or “unclear” risk of bias.

### Measures of treatment effect

The treatment effect for dichotomous outcomes (e.g., adverse events) will be expressed as a risk ratio (RR) with 95 % confidence intervals. Continuous outcomes will be expressed as mean differences (MD) with 95 % confidence intervals. Where continuous outcomes are measured using different scales, the treatment effect will be expressed as a standardized mean difference (SMD) with 95 % confidence interval (CI).

### Unit of analysis issues

The unit of analysis is each patient recruited in the studies, and we do not expect any crossover or cluster randomized trials.

### Dealing with missing data

Authors may be contacted in order to obtain supplemental information such as follow-up data.

### Assessment of heterogeneity

Heterogeneity among studies will be investigated by using the *χ*^2^ test and *I*^2^ test [14]. If significant heterogeneity is detected (*I*^2^ > 50 % or *p* < 0.1) for outcome measures, the calculations with a random effects model will be repeated using a random effects model as sensitivity analysis and we will consider results from both.

### Assessment of reporting bias

We plan to minimize the impact of reporting bias in our systematic review by ensuring a comprehensive search for eligible studies including three trial registries. A funnel plot and appropriate statistical tests for small study effects will be performed if ≥10 studies are available.

An attempt will be made to assess the possibility of publication bias utilizing the combined experience of the authors and their awareness of possible studies that were not published. The quality of evidence for all outcomes will be judged using the Grading of Recommendations Assessment, Development and Evaluation (GRADE) working group methodology [15, 16].

### Data synthesis

As it is known that there are a sufficient number of studies of similar design with adequate data, despite study protocol variations, it is expected that meta-analysis will be conducted on the identified studies. A random effects model will be used as primary analysis to estimate treatment effects. We will calculate pooled RRs, MDs, and SMDs and 95 % CIs across comparable studies using Review Manager (RevMan 5.3). When considerable heterogeneity (*I*^2^ > 80 %) is found between comparable studies, attempts will be made to explain heterogeneity.

### Subgroup analysis and investigation of heterogeneity

Subgroup analyses are planned for the following criteria:

- Ventilation status (invasive versus non-invasive respiratory support) at the time of entry into the study.
- Gestational age (<28 weeks, ≥28 weeks).
- Type of inhaled corticosteroid (budesonide, fluticasone, beclomethasone, others).
- Age at commencement of the intervention. Although prior Cochrane analyses separated studies into different analyses, the overlap between the so-called prevention and treatment is so broad that this variable may only be explored as a secondary analysis of interest.
- Dose and length of intervention.
- Different definitions of BPD (supplemental oxygen at 28 days and 36 weeks corrected gestational age, physiological definition).
- Mode of administration (metered-dose inhaler, nebulizer, others).

### Sensitivity analysis

We plan to perform sensitivity analyses based on assessment of risk of bias (evaluating only studies of low risk of bias for the primary outcome variables) and publication status (published studies only).

### Assessing the quality of evidence

We will use the GRADE approach to assess the quality of evidence for each outcome. We will judge the quality of evidence based on the suggested five criteria for downrating our confidence in effect estimates (risk of bias, inconsistency, imprecision, indirectness, and publication bias) and the three criteria for uprating our confidence (large effect, dose-response gradient, and opposing confounding). Based on these criteria, the quality of evidence judgment can range from very low to high.

## Discussion

Many treatments have been tried and tested for the prevention or treatment of BPD. Very few have been shown to be both safe and effective [17]. Clinicians anxiously await convincing evidence of any new intervention that may answer this urgent need. Previous systematic reviews and meta-analyses of inhaled corticosteroids for preterm infants have focused on specific indications and timing, although advancing understanding of the pathophysiology of BPD may suggest, for example, the distinction between prevention and treatment to be rather artificial.

This proposed review, by employing more inclusive inclusion criteria and adding new studies, aims to offer information that may be useful to the practicing neonatologist.

## Appendix

### Search Strategies

A provisional list of key words will include bronchopulmonary dysplasia, chronic lung disease of prematurity, corticosteroids, steroids, inhaled medications, inhalation, aerosols, infant-newborn, neonate, anti-inflammatory agents, dexamethasone, budesonide, beclomethasone dipropionate, flunisolide, and fluticasone propionate. Key words will be adjusted for each database as required and may be updated as required.

### Sample PubMed search

(bronchopulmonary dysplasia OR chronic lung disease OR chronic lung disease of prematurity) and (corticosteroids or steroids or anti-inflammatory agents) and (dexamethasone or budesonide or beclomethasone dipropionate or flunisolide or fluticasone propionate) and (infant-newborn or neonate) and (inhaled medications or inhalation or aerosols)

((“bronchopulmonary dysplasia”[MeSH Terms] OR (“bronchopulmonary”[All Fields] AND “dysplasia”[All Fields]) OR “bronchopulmonary dysplasia”[All Fields]) OR (chronic[All Fields] AND (“lung diseases”[MeSH Terms] OR (“lung”[All Fields] AND “diseases”[All Fields]) OR “lung diseases”[All Fields] OR (“lung”[All Fields] AND “disease”[All Fields]) OR “lung disease”[All Fields])) OR (“bronchopulmonary dysplasia”[MeSH Terms] OR (“bronchopulmonary”[All Fields] AND “dysplasia”[All Fields]) OR “bronchopulmonary dysplasia”[All Fields] OR (“chronic”[All Fields] AND “lung”[All Fields] AND “disease”[All Fields] AND “prematurity”[All Fields]) OR “chronic lung disease of prematurity”[All Fields])) AND ((“adrenal cortex hormones”[Pharmacological Action] OR “adrenal cortex hormones”[MeSH Terms] OR (“adrenal”[All Fields] AND “cortex”[All Fields] AND “hormones”[All Fields]) OR “adrenal cortex hormones”[All Fields] OR “corticosteroids”[All Fields]) OR (“steroids”[MeSH Terms] OR “steroids”[All Fields]) OR (“anti-inflammatory agents”[Pharmacological Action] OR “anti-inflammatory agents”[MeSH Terms] OR (“anti-inflammatory”[All Fields] AND “agents”[All Fields]) OR “anti-inflammatory agents”[All Fields] OR (“anti”[All Fields] AND “inflammatory”[All Fields] AND “agents”[All Fields]) OR “anti-inflammatory agents”[All Fields])) AND ((“dexamethasone”[MeSH Terms] OR “dexamethasone”[All Fields]) OR (“budesonide”[MeSH Terms] OR “budesonide”[All Fields]) OR (“beclomethasone”[MeSH Terms] OR “beclomethasone”[All Fields] OR (“beclomethasone”[All Fields] AND “dipropionate”[All Fields]) OR “beclomethasone dipropionate”[All Fields]) OR (“flunisolide”[Supplementary Concept] OR “flunisolide”[All Fields]) OR (“fluticasone”[Supplementary Concept] OR “fluticasone”[All Fields] OR “fluticasone propionate”[All Fields])) AND ((“infant, newborn”[MeSH Terms] OR (“infant”[All Fields] AND “newborn”[All Fields]) OR “newborn infant”[All Fields] OR (“infant”[All Fields] AND “newborn”[All Fields]) OR “infant newborn”[All Fields]) OR (“infant, newborn”[MeSH Terms] OR (“infant”[All Fields] AND “newborn”[All Fields]) OR “newborn infant“[All Fields] OR “neonate”[All Fields])) AND (((“inhalation”[MeSH Terms] OR “inhalation”[All Fields] OR “inhaled”[All Fields]) AND (“pharmaceutical preparations”[MeSH Terms] OR (“pharmaceutical”[All Fields] AND “preparations”[All Fields]) OR “pharmaceutical preparations”[All Fields] OR “medications” [All Fields])) OR (“inhalation”[MeSH Terms] OR “inhalation”[All Fields]) OR (“aerosols”[MeSH Terms] OR “aerosols”[All Fields])) AND (Clinical Trial[ptyp] AND “humans”[MeSH Terms]) = 33.

## Abbreviations

BPD: bronchopulmonary dysplasia
ELBW: extremely low birth weight
IVH: intraventricular hemorrhage
PMA: postmenstrual age
PNA: postnatal age
PRISMA: Preferred Reporting Items for Systematic Reviews and Meta-Analyses
RCT: randomized controlled trial
ROP: retinopathy of prematurity
